# Recipient-Specific Risk Factors Impairing Patient and Graft Outcome after Pediatric Liver Transplantation—Analysis of 858 Transplantations in 38 Years

**DOI:** 10.3390/children8080641

**Published:** 2021-07-27

**Authors:** Christoph Leiskau, Norman Junge, Eva-Doreen Pfister, Imeke Goldschmidt, Frauke Mutschler, Tobias Laue, Johanna Ohlendorf, Hamoud Nasser, Jan Beneke, Nicolas Richter, Florian Vondran, Ulrich Baumann

**Affiliations:** 1 Pediatric Gastroenterology, Hepatology and Liver Transplantation, Department of Pediatric Kidney, Liver and Metabolic Diseases, Hannover Medical School, 30625 Hannover, Germany; junge.norman@mh-hannover.de (N.J.); pfister.eva-doreen@mh-hannover.de (E.-D.P.); Goldschmidt.imeke@mh-hannover.de (I.G.); mutschler.frauke@mh-hannover.de (F.M.); laue.tobias@mh-hannover.de (T.L.); ohlendorf.johanna@mh-hannover.de (J.O.); nasser.hamoud@mh-hannover.de (H.N.); Baumann.U@mh-hannover.de (U.B.); 2 Pediatric Gastroenterology, Department of Pediatrics and Adolescent Medicine, University Medical Centre Göttingen, Georg August University Göttingen, 37073 Göttingen, Germany; 3 Core Facility Quality Management and Health Technology Assessment in Transplantation, Hannover Medical School, 30625 Hannover, Germany; beneke.jan@mh-hannover.de; 4 Department of General, Visceral and Transplant Surgery, Hannover Medical School, 30625 Hannover, Germany; richter.nicolas@mh-hannover.de (N.R.); Vondran.Florian@mh-hannover.de (F.V.)

**Keywords:** pediatric liver transplantation, long-term outcome, patient survival, graft survival, recipient-specific variables, independent risk factors, multivariate analysis, portal vein thrombosis

## Abstract

(1) Background and Aim: Despite excellent long-term results in pediatric liver transplantation (pLTx), mortality and graft loss still are to be diminished. We aim to describe time-dependent changes and long-term outcome of a large single-center pLTx cohort and to identify independent recipient-related risk factors impairing patient and graft survival. (2) Methods: This is a retrospective single-center study analyzing all pediatric liver transplants from 1983–2020. Risk factors for mortality and graft loss were identified by univariable and multi-linear regression analysis. (3) Results: We analyzed 858 liver transplantations in 705 pediatric patients. Five-year patient/graft survival increased from 60.9%/48.0% (1983–1992) to 97.5%/86.5% (OR = 12.5; *p* < 0.0001/OR = 6.5; *p* < 0.0001) (2014–2020). Indications changed significantly over time, with a higher proportion of patients being transplanted for malignancies and metabolic disease and indications of PFIC and α1AT-deficiency declining. The era of transplantation (log7.378/9.657; *p* < 0.0001) and indication of acute liver failure (log = 1.944/2.667; HR = 2.015/1.772; *p* = 0.0114/0.002) impairs patient/graft survival significantly in the multivariate analysis. Furthermore, patient survival is worsened by re-transplantation (log = 1.755; HR = 1.744; *p* = 0.0176) and prolonged waiting times in high-urgency status (log = 2.588; HR = 1.073; *p* = 0.0026), whereas the indication of biliary atresia improved outcome (log = 1.502; HR = 0.575; *p* = 0.0315). Graft survival was additionally impaired by pre-existing portal vein thrombosis (log = 1.482; HR = 2.016; *p* = 0.0330). (4) Conclusions: Despite more complex indications, patient and graft survival after pLTx continue to improve.. Acute liver failure remains the indication with poorest outcome, and listing for high urgency liver transplantation should be considered carefully and early to keep waiting time on HU list short. Furthermore, pre-transplant portal vein thrombosis should be prevented whenever possible to improve graft survival.

## 1. Introduction

(Pediatric) liver transplantation is inevitably connected to Prof. T.E. Starzl, who performed the first orthotopic liver transplantation in 1963 [1] and the first successful liver transplantation in 1967 [2], both in children with biliary atresia—a disease leading unavoidably to death by biliary cirrhosis in those days. More than 50 years later, pediatric liver transplantation (pLTx) is a well-established procedure for children with chronic or acute liver failure and liver-based metabolic disease and has 5-year survival rates exceeding 90% in the experienced pLTX-centers [3]. The invention of split liver transplantation by Rudolf Pichlmayr in Hannover in 1988 [4], living-related liver donation, and the introduction of the PELD (pediatric end-stage liver disease) Score as allocation criteria, as well as the continuous improvement of immunosuppressive strategies, have contributed highly to the comparatively good outcome in children after liver transplantation. Furthermore, there is a comparatively low mortality of children on the transplant waiting list, even in times of organ shortage and even more aggravated during the SARS-CoV2-pandemic [5]. Even though our focus must also lie on a good long-term outcome, with the least possible impact on quality of life [3], the avoidance of graft loss and worst of all the mortality of liver recipients are still the primary goals of clinicians attending pediatric liver transplantation.

Risk factors for impaired patient survival after pediatric liver transplantation have been analyzed in several studies, but, at least partially, with controversial conclusions. Consistently, transplantation in earlier years/eras was found to be a risk factor [6,7,8], as was re-transplantation [8,9,10,11,12]. Some researchers found low patient weight/age to be a risk factor [8,11], whereas others found smaller/younger patients to be better suited for graft and patient survival [13]. Mostly, acute liver failure as an indication for pLTx was found to be a risk factor [7,14], some studies also found malignant diseases to have a higher risk of mortality [7]. Further, ICU-treatment prior to transplantation [7,8,10] and pre-existing renal damage [7,8,11,15] impaired post-Tx patient survival. AB0-incompatible transplantation was a risk factor for impaired patient and graft survival in a huge cohort of pediatric LTx-patients with living-related transplantation [14]. One study found pre-existing portal vein thrombosis to be a risk factor for mortality after pLTx, the influence on graft survival was not analyzed [16].

Risk factors for impaired graft survival have been described as the era of transplantation [15]. Some researchers also found very young and comparatively older children at risk for early re-transplantation. [17,18] Diagnosis of acute liver failure also showed a risk for early graft loss [18] and overlapping transplantation in high urgency.

Most of the previously discovered risk factors are given and not to be modified; our aim was to identify ideally modifiable recipient-specific risk factors for impaired graft and patient survival in order to create the optimal, baseline conditions for patient and graft survival. Furthermore, this is the first study to describe the long-term outcome of our pediatric liver transplant cohort and the largest European single-center pediatric liver transplant cohort described so far. The first liver transplantation was performed at Hannover Medical School in 1972, the first pediatric liver transplant in 1979 and a regular pediatric liver transplant program was set up in 1983. With more than 850 pediatric liver transplants in total and an actual annual rate of around 30 pLTX, Hannover Medical School is one of the largest pLTX programs in Europe. We aimed to detect changes over time, regarding indications and outcome of pLTx recipients.

## 2. Patients and Methods

### 2.1. Study Design and Setting

This is a single center (EUROTRANSPLANT region) retrospective cohort study analyzing the data of all consecutive liver transplants in pediatric patients (<18 years at date of pLTx) from 1983 to 2020. 

All transplantations took place at our center and were performed by the department of visceral and transplant surgery and medically attended by the department of pediatric hepatology and the pediatric intensive care unit.

### 2.2. Inclusion and Exclusion Criteria

For the main analysis, all pediatric liver transplantations were included; no pediatric liver transplant recipients were excluded. Three patients in our cohort who had already reached adulthood at the time of transplantation while the transplantation was still performed under pediatric care were excluded. Sub-analyses were performed within the group of primary transplantations. For sub-analyses, patients were divided into pediatric age groups (infants < 1 year, preschoolers 1–5 years, school children 6–11 years and adolescents 12–17 years of age) and eras of transplantation (5 eras with approximately the same patient size, and different time spans). 

### 2.3. Study Outcome Parameters

The main outcome parameters were patient survival (endpoint: death after pediatric liver transplantation), graft survival (endpoint: loss of graft due to re-transplantation or death of the patient).

All patients were followed up from the date of transplant until either death (*n* = 141) or the date of last known follow-up (*n* = 564). Follow-up was performed by screening the documentation of visits in pediatric and adult hepatology clinics and, in case of patients being lost to follow-up data were gained from population registries. Median follow-up period after transplantation was 10.16 years (0.03–37.88 years; IQR 3.6–18.5 years); adding up to 8547 years at risk after pLTx.

### 2.4. Data Retrievement

Data regarding the organ recipients were gained by revision of surgery reports and discharge letters, the files of the patients, our electronic patient database and the laboratory data storage program ALIDA. Data regarding the organ donors was retrieved by EUROTRANSPLANT and our patient database, in cases of living-related donation.

Data were collected retrospectively and stored in a JMP Pro (SAS Institute Inc., Cary, NC, USA) table. Statistics were calculated in JMP (Version 14 pro). 

### 2.5. Ethical Considerations

All parents/caregivers of patients analyzed in this study provided informed consent that their children’s data were allowed to be used for scientific purposes. Patient data were anonymized prior to analysis. Ethics counselling was not necessary because of the retrospective design of the study, according to the guidelines of our Ethics’ Committee.

### 2.6. Statistical Methods

Continuous data are represented in this study as median, interquartile range, and minimum and maximum, whereas binary variables are represented as positive frequencies (*n*) and percentages (%) within analyzed groups of patients. The Wilcoxon test was used to compare continuous data between groups and the two-sided Pearson’s Chi-squared test was used to compare the proportions of binary variables between groups. 

Risk factors for patient and graft survival and their significance were calculated by univariable regression analysis (time to event: patient/graft survival, censor: death after transplantation/graft loss due to re-transplantation or death of the recipient). Factors with *p*-values ≤ 0.250 in univariable logistic regression (based on the Wald test) were considered for inclusion into multivariable regression modeling. Final multivariable regression models were achieved by gradual reverse probability elimination of insignificant variables until only significant variables (*p* < 0.05) remained in the final logistic regression models.

For continuous variables, hazard ratios were calculated by Cox regression analysis and displayed per change of unit in regression.

Changes in the percentage of specific indications over time were analyzed by nominal logistic regression analysis, odds ratios are displayed for comparison of Era 1 to Era 5.

Odds ratios (OR) and hazard ratios (HR) and their respective 95-confidence intervals (95%-CI) are provided for uni- and multivariate logistic and Cox regression analysis results, respectively. Survival rates of different transplant eras were compared by nominal logistic regression analysis.

## 3. Results

### 3.1. Baseline Characeristics

From 1 January 1983 to 31 December 2020, 858 orthotopic liver transplantations were performed in 705 pediatric patients (i.e., 705 primary transplants, 133 secondary, 19 tertiary-LTx and 1 quartenary LTx). 

#### 3.1.1. Indications for Pediatric Liver Transplantation

The main indications for primary liver transplantation were biliary atresia (more than one third of the patients), acute liver failure (12%), PFIC and Alagille syndrome, metabolic and malignant diseases, alpha-1-AT-deficiency, Wilson’s disease, PSC and autoimmune liver disease (Table 1). The Kasai procedure was performed in 94.2% of patients transplanted for biliary atresia.

Main indications for re-transplantation were vascular complications, chronic graft failure, primary non-function of the graft, biliary complications, and chronic rejection.

#### 3.1.2. Percentage of Indications Related to Era of Transplantation

Over time, analysis of the indications for primary liver transplantation related to the eras, shows a significant change of indications in our center. Whereas the percentage of patients being transplanted for biliary atresia (32% in Era 1, maximum of 47.6% in Era 3 to 31% in Era 5) did not show a clear tendency, transplantation for PFIC (14.3% to 6.9%; OR 0.448; 95%CI = 2.202–0.995; *p* = 0.0486) and α1AT-deficiency (6.4% to 0.7%; OR 0.102; 0.013–0.815; *p* = 0.0313) significantly decreased. The percentage of patients being transplanted for acute liver failure (17.7% in Era 1 to 9.7% in Era 5) and Alagille syndrome (5% to 2%) receded, too, but did not reach a level of significance. 

There is a significant increase of patients being transplanted for metabolic diseases (5% in Era 1 to 13.8% in Era 5; OR 3.063, 95%CI:1.252–7.493; *p* = 0.0142) and malignant diseases (2% in Era 1 to 11% in Era 5, OR 5.705; 1.624–20.039; *p* = 0.0066). The percentage of patients with cystic fibrosis also increased (0% to 7.6%), but due to the small patient numbers could not be statistically analyzed (Table 2).

#### 3.1.3. Recipient Specific Baseline Characteristics

Median age at time of liver transplantation was 4.02 years (0.05–17.98 years; IQR 1.15–10.47), while mean age was 6.03 years, showing the non-Gaussian distribution of the age at transplant. Nearly 40% of the children were transplanted within the first two years of age. Gender distribution was nearly even with 413 girls (48.1%). Mean overall time on waiting list, irrespective of status, was 158.9 days, with 199.5 days in non-high-urgency status and 6.2 days in high urgency status. Pre-transplant portal vein thrombosis was present in 21 (3.6%) of the children. All baseline characteristics can be found in Table 3.

### 3.2. Patient Survival

**Figure 1:**
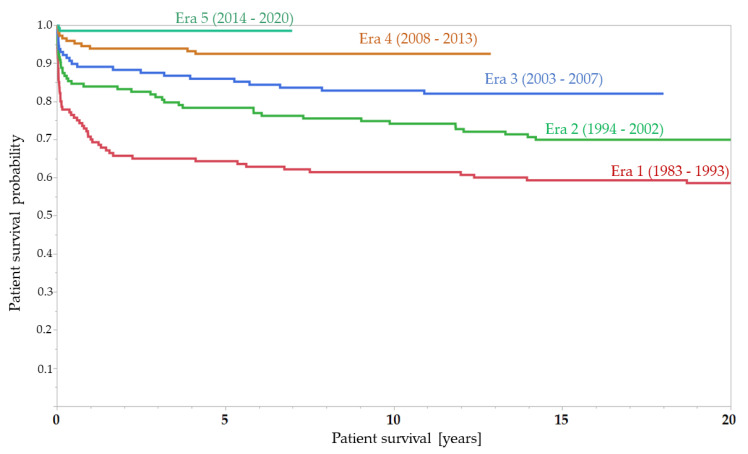
Kaplan–Meier-curve for patient survival (by era of transplantation). Note that in Era 5, only 56 out of 145 patients have completed the 5-year surveillance.

**Figure 2:**
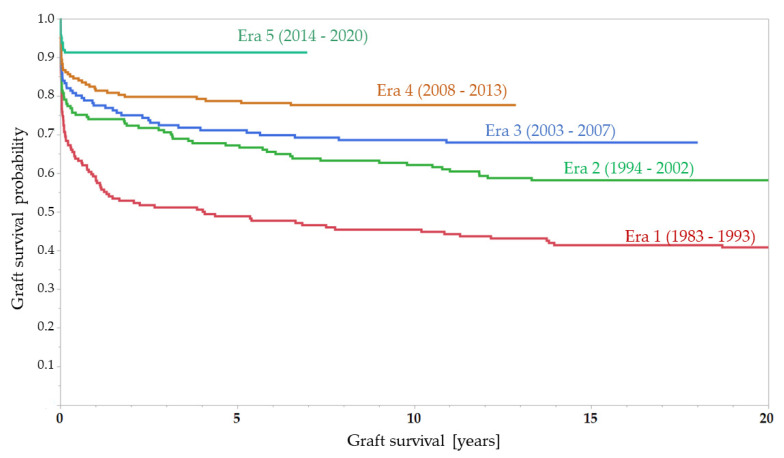
Kaplan–Meier-curve for graft survival (by era of transplantation). Note that in Era 5, only 59 out of 162 grafts have completed the 5-year surveillance.

Overall, for the whole group, 1-year patient survival was 84.6%, 5-year-survival 79.2%, 10-year survival 72.8%, 20-year survival 62.5%. As shown in the Kaplan–Meier-curve (Figure 1 and Figure 2), patient survival has improved significantly in recent years, with a 5-year-survival rate from 60.9% in Era 1 to 97.5% in the recent era (OR 12.0; 95%CI 3.6–39.8; *p* < 0.001); 10 year survival rose from 58.0% in Era 1 to 89.2% in Era 4 (OR 6.0; 95%CI 2.9–12.3; *p* < 0.001), whereas 20-year survival improved from 54.6% in Era 1 to 72.6% in Era 2 (OR 2.2; 1.4–3.6, *p* = 0.0013). Of all patients who died after transplantation (141/20.6%), more than two-thirds (103/14.4%) were transplanted in the two early eras and only 3 (1.14%) were transplanted in the most recent era, while group sizes in all eras were similar. As the oldest patient was born in 1967, no natural death would be expected, so far.

### 3.3. Cause of Death of the Pediatric Liver Recipients

The main reasons for recipient mortality (total *n* = 141, 20.0%) after pLTx were infection (43.9%), cerebral events (14.4%), graft dysfunction (12.2%) and malignancies (de novo/recurrence) (9.4%), as shown in Table 3. 

### 3.4. Graft Survival

Graft survival showed a similar improvement over the years: Altogether, 285 cases of graft loss (33.1%) were observed. The main reasons for graft loss were death of the recipient (mostly caused by infection) (21.4%), vascular complications (17.5%), initial non-function of the graft (15.6%), chronic rejection (9.8%), and chronic graft failure (5.6%).

Overall, 5-year-graft-survival was 68.1%, with a significant increase from 48.0% in Era 1 to 86.5% in Era 5 (OR 6.9; 95%CI 2.9–16.4; *p* < 0.0001); 20-year-graft-survival also improved significantly from 39.4% in Era 1 to 58.3% in Era 2 (OR 2.1; 95%CI 1.3–3.5; *p* = 0.0017).

### 3.5. Recipient-Specific Risk Factors of Influence on Patient Survival after Transplantation 

#### 3.5.1. Recipient-Specific Factors of Influence on Patient Survival (Univariate Analysis)

Univariable proportional hazard analysis for patient survival revealed risk factors associated with premature death after liver transplantation (Table 4). A higher recipient age at transplantation was associated with significantly worse outcome (1.038 per change in unit of regression, 1.009–1.068; *p* = 0.017), in keeping with this, adolescent age at time of transplantation (age group > 12–18 years) was another risk factor for impaired survival (1.627, 1.120–2.322; *p* = 0.014) and infants were at lowest risk (0.645; *p* = 0.048).

Children transplanted for acute liver failure had a significantly higher risk of mortality after pLTx (HR 2.536, 1.729–3.615; *p* < 0.001), whereas transplantation for biliary atresia (0.629; 0.441–0.878; *p* = 0.006), PFIC (0.457; 0.194–0.899; *p* = 0.021) had a significantly better outcome. Other indications did not have a significant impact on survival in our cohort. Re-transplantation was significantly associated with impaired patient survival (HR 1.781, *p* = 0.0010). 

Pre-existing portal vein thrombosis did not have a significant influence on patient survival.

Recipient’s total bilirubin at the time of pLTx correlated negatively with survival (1.002; 1.000–1.003; *p* = 0.004), as with an elevated INR (1.673; 1.167–2.285; *p* = 0.0007). 

The era of transplantation had a significant impact on patient survival, as children transplanted in era 1 (HR 3.186, *p* < 0.001) and era 2 (HR 1.466; *p* = 0.045) had a significantly higher risk of premature death, whereas children transplanted in era 4 and 5 had a significantly better chance of survival (HR 0.347; *p* < 0.001 and HR 0.073, *p* < 0.001, respectively). The factor of being on a high-urgency waiting list for liver transplantation was significantly associated with impaired patient survival (HR 2.160; *p* < 0.00012). Longer waiting times on the transplant list, independent of the waiting list status, was positively correlated with survival (HR 0.998; *p* = 0.002), whereas a waiting time in T status did not have a significant influence and a prolonged waiting time on the high-urgency list led to a higher risk of premature death after pLTx (HR 1.057; *p* = 0.009). The Lab-MELD-Score was not significantly associated with post-Tx survival, whereas an increased pediatric MELD-score was associated with better survival after transplantation.

Biliary anastomosis during liver transplantation via biliodigestive anastomosis was associated with better survival (0.735; 0.546–0.994; *p* = 0.046).

A longer duration of the post-pLTx ICU stay had a significant, negative influence on patient survival (1.010; 1.004–1.017; *p* = 0.004), whereas a prolonged non-ICU in-patient stay was correlated with improved survival (0.960; 0.952–0.968, *p* < 0.0001).

The need for re-transplantation was highly correlated to impaired survival (HR 1.934; 1.400–2.637; *p* < 0001).

#### 3.5.2. Independent Recipient-Specific Factors of Influence on Patient Survival after Transplantation (Multivariate Analysis)

Multivariate analysis showed that era of transplantation (log 7.378; *p* < 0.0001), length of waiting time on the high-urgency list (2.588; HR 1.073; *p* = 0.0109), diagnosis of acute liver failure (1.944; HR2.015; *p* = 0.0114), and subsequent re-transplantation (1.755, HR1.744; *p* = 0.0176) were independent and highly significant risk factors for impaired patient survival (Table 5). Diagnosis of biliary atresia significantly improved the outcome in the multivariate regression (log 1.502; HR 0.575; *p* = 0.0315).

### 3.6. Risk Factors Influencing Graft Survival

#### 3.6.1. Recipient-Specific Factors Influencing Graft Survival (Univariate Analysis)

Age as a continuous variable did not influence graft survival, but the age group of school children 6–12 years (HR:0.704; 95%CI:0.510–0.951; *p* = 0.0214) were significantly less often affected by graft loss (Table 4).

Increased recipient INR was a risk factor for graft loss (1.370; 1.085–1.682; *p* = 0.0096).

Diagnosis of acute liver failure was a risk factor for impaired graft survival (HR:2.046; 1.464–2.790; *p* < 0.0001), regardless of the reason for graft loss, whereas a diagnosis of cystic fibrosis was significantly associated with better graft survival (HR:0.413; 0.147–0.896; *p* = 0.0225). 

The era of transplantation significantly affected graft survival, irrespective of definition of graft loss, with transplantation in era 1 being a risk factor for graft loss (HR = 2.552; 1.997–3.245; *p* < 0.0001), while pLTx in era 4 (0.584; 0.415–0.803; *p* = 0.0007) and era 5 (0.246; 0.137–0.406; *p* < 0.0001) led to better graft survival.

Prolonged post-transplant ICU stay impaired graft survival (HR 1.009; 1.003–1.014; *p* = 0.0029), whereas a longer non-ICU inpatient stay improved graft survival.

#### 3.6.2. Multivariate Analysis for Independent Factors Influencing Graft Survival

Independent, significant risk factors for impaired graft survival by multivariate analysis were, early era of transplantation (log 9.657; *p* < 0.0001), underlying diagnosis of acute liver failure (log 3.923; HR 1.935; *p* = 0.0001), and pre-existing portal vein thrombosis (log 1.482; HR 2.016; *p* = 0.0330) (Table 6).

## 4. Discussion

This is the first study to describe the complete pediatric liver transplant cohort at our center and the largest European single center pLTx cohort. 

### 4.1. Patient and Graft Survival

Overall, patient survival is comparable to pediatric liver transplantation cohorts in other experienced centers, the one- and 5-year patient survival of 97.5% in the most recent era is slightly higher when compared to the recently published reports of the North American SPLIT registry [19], documenting a similar time span, whereas 5-year-graft survival is slightly lower when compared to the data from North America. The significant improvement of 5-year-survival over the years is comparably documented by all experienced centers.

### 4.2. Changes of Indications for Pediatric Liver Transplantation over Time

Analysis of the indications for primary liver transplantation related to the eras, shows a significant shift of the percentual share of transplant indications in our center from some of the “classic” indications for pediatric liver transplantation, such as PFIC and a1AT-deficiency, to more complicated, often multisystemic indications such as metabolic diseases and malignancy.

The change of indications over time has also been documented by other authors, McKiernan et al., documented a decrease in patients being transplanted for a1-AT-deficiency and Wilson’s disease, while the percentage of patients being transplanted for metabolic disease, without structural liver damage, was rising in the north American registry, which supports our findings [20]. Other studies also noted an increase in patients being transplanted for hepatoblastoma and a decrease in transplantations for acute liver failure [21]. However, total percentage of patients transplanted for malignant disease in the recent era (11%) is clearly higher than in multicenter studies [21], indicating a specialization in our center. The decrease in patients being transplanted with PFIC may be related to earlier and more precise genetic diagnostics and novel treatments, possibly postponing transplantation [22].

### 4.3. Recipient-Specific Variables Influencing Patient Survival

The indication for liver transplantation had a significant impact on patient survival in our study—patients with acute liver failure being at higher risk of mortality after pLTx, which is consistent with most studies [7,14,23,24], whereas biliary atresia and PFIC had a favorable outcome—the former is also described in other studies [11,21]. In multivariate analysis, acute liver failure remained the second most important independent risk factor for mortality and transplantation for biliary atresia proved to be an independent factor associated with improved patient survival. Biliary atresia in its non-syndromic form is limited to the liver and can be cured by liver transplantation; the need for transplantation, in most cases, is foreseeable and can be timed to allow for pLTx in a stable state in many patients. Patients with acute liver failure are often transplanted in very poor condition, sometimes with the need to accept marginal donors—all of which leading to a high mortality rate after pLTx.

Regarding the age at transplantation, our study showed higher age to be a risk factor for mortality, patients transplanted in infancy had a significantly better outcome, whereas transplantation in adolescence was a risk factor for premature death. This stands in contrast to most studies, which—if at all—have found early infancy to be a risk factor for impaired survival [6,8,11,25,26], or seen no influence at all [27]. One study found superior results for patient and graft survival in small infants [13], and two studies also found older patients at a higher risk of mortality [25,28]. The better outcome of infants in our center may be related to comparably longer experience in partial and split liver transplantation in our center, both in surgical and pediatric care. However, as age as a risk factor lost its significance in the multivariate analysis, we assume a bias due to the fact that a huge percentage of patients were transplanted in infancy for biliary atresia which is associated with a comparably better outcome.

A higher INR and bilirubin level at the time of transplantation was also associated with an inferior outcome, in keeping with other studies [11] and clinically comprehensible, as very sick patients or patients with acute or chronic liver failure have a worse outcome and more perioperative complications than patients transplanted in a relatively stable state—however, these risk factors could not be reproduced in multivariate analysis.

The waiting list status and waiting time had an influence on patient survival, with patients being listed in high urgency status at higher risk of mortality, especially with a prolonged waiting time with the latter being reproducible as an independent risk factor in multivariate analysis. The association of status-independent prolonged waiting time with improved patient survival was biased by the fact that the waiting time for patients in HU status was generally shorter and these patients had a higher risk of mortality.

The era of transplantation was highly and significantly associated with patient survival; remarkably, this improvement continues up to the most recent era, even when compared to the era around 2010 (but not on significant level). As we have included patients transplanted until the end of 2020 in the recent era, only 56 of 162 patients (34.6%) have completed the 5-year-surveillance. Advancements in surgical techniques, pediatric intensive care and pre- and post-transplant pediatric hepatology attendance, immunosuppressive therapy, and screening for known complications, improve survival after pLTx. The era of transplantation was also the most significant and highest correlated factor in multivariate analysis, which supports the findings of many colleagues [6,25].

Re-transplantation was a highly significant risk factor for impaired patient survival in uni- and multivariate analysis, being another critical procedure in potentially instable patients, as seen in many other studies [8,10,11,12,29].

The length of post-transplant ICU-stay, indicating a more complicated course, also led to a higher mortality risk.

### 4.4. Recipient-Specific Variables Influencing Graft Survival 

The analysis of factors influencing graft survival revealed a lower risk for graft loss in the group of patients between 6 and 12 years of age. As age groups have been defined differently, a direct comparison is difficult, but some other studies have seen this age group rather as a risk factor for an unfavorable outcome [18,28] and liver recipients in infancy at a higher risk of graft loss [30]—which we could not reproduce in our analysis.

Regarding indications, a diagnosis of acute liver failure was not only associated with impaired patient survival, but also an independent risk factor for impaired graft survival, as seen by other researchers [14,24] in uni- and multivariate analysis. This can possibly be explained by the comparatively high percentage of deceased patients after pLTx with ALF, accounting for many graft losses due to the patient’s death; but in our data, ALF, as a negative predictor, remained significant, even if only graft loss due to re-transplantation was considered.

Interestingly, we found cystic fibrosis and hepatoblastoma to be indications with improved graft survival, but these indications lost their significance in multivariate modeling, probably due to confounders—we have not found comparative data in other studies, except for another study reporting a small cohort of liver-transplanted CF patients without graft loss [31]. CF patients are transplanted at comparatively older ages and the possibility of a direct biliary anastomosis may lead to improved graft survival. Further, these patients are under very close medical attendance due to their multisystemic chronic condition.

As in most previous studies, the era of transplantation had a highly significant impact on graft survival in univariate and multivariate analysis, underlining the impact of the advances in surgical and medical care, enabling long-term graft survival [8,25].

Pre-existing portal vein thrombosis before liver transplantation was an independent and significant risk factor for graft loss in the multivariate analysis in our study. Obviously, with a pre-existing thrombosis one might suspect a worse portal vein flow, with possible post-transplant PV thrombosis. To our knowledge, there is only one study in a pediatric cohort that found pre-Tx-PV-thrombosis to be a risk factor for mortality (which we could also see, but not on a significant level), and a study in adult patients that found it to be a risk factor for graft loss, but interestingly by hepatic artery thrombosis post-transplant [32]. In our patients with pre-existing portal vein thrombosis, death of the patient (infection (4), de novo malignancy (1), GI-bleeding (1) and ARDS (1)), chronic graft failure (2), initial non-function (2) and portal vein thrombosis (1) were the reasons for graft loss, no cases of hepatic artery thrombosis were observed.

### 4.5. Limitations and Strengths of the Study

Due to the retrospective design of this study, we had to deal with some missing data which could not be retrieved retrospectively, especially regarding pre-transplant laboratory variables of the recipients, which limits the explanatory power of our data.

Data regarding graft loss or re-transplantation may be incomplete, as some patients are no longer under the care of our center and, in most cases, we do not have access to the patients’ data who had a possible re-transplantation in another center. In the follow-up, we could retrieve data regarding survival of patients no longer under our care from population registries, but in cases of mortality we have no information regarding the cause of death.

Due to the single-center design of the study, some observed developments, especially regarding changes in indications over time, may be center-specific effects that would need to be validated in bigger, multi-center cohort studies.

However, with more than 850 pediatric liver transplantations in more than 700 patients this is one of the largest pediatric single-center studies and to our knowledge the largest European single-center cohort described so far. We have gathered pre-, peri-, and post-transplant data comprising nearly four decades of pediatric liver transplantation in which major developments and new treatment options have been established and identified changes and influencing factors in this large cohort.

## 5. Conclusions

Our analysis of independent recipient-specific risk factors for patient and graft survival after pediatric liver transplantation has supported the results of previous analyses, but also led to new findings. Patient and graft survival improved continuously over the years and has reached an excellent level in the recent era. We see a significant change in indications from “classic” pediatric indications (PFIC, alpha-1-antitrypsin-deficiency, Alagille syndrome) to more complex diagnoses of malignant and metabolic diseases.

In multivariate analysis, early era of transplantation and the diagnosis of acute liver failure impair patient and graft survival independently and significantly. Further, patient survival was better in patients with biliary atresia. Prolonged waiting time in high urgency status for HU patients was a risk factor for mortality. For graft survival, we found pre-existing portal vein thrombosis to be an additional, independent risk factor. Many of the risk factors can hardly be modified, but as clinicians working in the field of pediatric liver transplantation, we should lay our focus on the factors that can, at least partially, be influenced: careful and early choice of patients needing high urgency transplantation and prevention of portal vein thrombosis, often a careful balancing act between diuretics and fluid intake, especially in end-stage liver disease patients with ascites. Further multicenter studies validating the impact of the risk factors identified in our study would be desirable.

## Figures and Tables

**Table 1:** Indications for primary and re-transplantation.

INDICATIONS for Pediatric Liver Transplantation	*n*	%
PRIMARY TRANSPLANTATIONS	705	100
Biliary atresia	259	36.7
Kasai-Op in case of BA (*n* = 259)	244	94.2
Acute liver failure	82	11.6
PFIC	60	8.5
Cystic fibrosis	32	4.5
Alagille syndrome	30	4.3
Hepatoblastoma	29	4.1
Alpha-1-antitrypsin-deficiency	26	3.7
Wilson’s disease	18	2.6
Primary sclerosing cholangitis	16	2.3
Metabolic disease (other)	13	1.8
Autoimmune hepatitis	12	1.7
Urea Cycle Disorder	12	1.7
ARPKD	11	1.6
Hyperoxaluria	11	1.6
Malignoma other than Hepatoblastoma	8	1.1
Tyrosinemia	6	0.9
GALD	5	0.7
Crigler-Najjar syndrome	4	0.6
Viral hepatitis-related cirrhosis	4	0.6
Cryptogenic biliary or other cirrhosis	34	4.8
Other vascular or biliary liver disease	27	3.8
RE-TRANSPLANTATIONS	153	100
Secondary Transplantation	133	86.9
Tertiary Transplantation	19	12.4
Quartenary Transplantation	1	0.7
Re-PLTx: vascular complications	58	37.9
Re-PLTx: chronic graft failure	42	27.5
Re-PLTx: primary non-function	34	22.2
Re-PLTx: biliary complications	11	7.2
Re-PLTx: chronic rejection	8	5.2

**Table 2:** Change of indications for primary pediatric liver transplantation over time.

Indication for Primary Pediatric Liver Transplantation	Number of Patients (% of pLTx in Resp. Era)				
	Era 1 (1983–1993)	Era 2 (1994–2002)	Era 3 (2003–2007)	Era 4 (2008–2013)	Era 5 (2014–2020)
Acute liver failure	24 (17.1%)	18 (12.6%)	14 (10.9%)	16 (10.8%)	15 (10.3%)
AIH/PSC	3 (2.1%)	6 (4.2%)	3 (2.3%)	6 (4.1%)	2 (1.4%)
Alagille syndrome	7 (5.0%)	8 (5.6%)	7 (5.5%)	5 (3.4%)	3 (2.1%)
α1-antitrypsin-deficiency	9 (6.4%)	8 (5.6%)	4 (3.1%)	4 (2.7%)	1 (0.7%)
Biliary atresia	45 (32.1%)	52 (36.4%)	61 (47.7%)	56 (37.8%)	45 (31.0%)
Cryptogenic cirrhosis	13 (9.3%)	10 (7.0%)	7 (5.5%)	17 (11.5%)	14 (9.7%)
Cystic fibrosis	0	4 (2.8%)	9 (7.0%)	8 (5.4%)	11 (7.6%)
Malignoma	3 (2.1%)	4 (2.8%)	1 (0.8%)	13 (8.8%)	16 (11.0%)
Metabolic disease	7 (5.0%)	10 (7.0%)	0	6 (4.1%)	20 (13.8%)
Other	4 (2.9%)	7 (4.9%)	9 (7.0%)	1 (0.7%)	2 (1.4%)
PFIC	20 (14.3%)	11 (7.7%)	8 (6.3%)	11 (7.4%)	10 (6.9%)
Wilson’s disease	3 (2.1%)	2 (1.4%)	5 (3.9%)	4 (2.7%)	4 (2.8%)
Total	140	143	128	148	145

**Table 3:** Recipient baseline characteristics.

Categorical Variables	*n*	%
PEDIATRIC AGE GROUP		
Age group < 1 years	193	22.5
Age group ≥ 1 < 6 years	310	36.1
Age group ≥ 6 < 12 years	184	21.4
Age group ≥ 12 years	171	19.9
Recipient female	413	48.1
Sex mismatch	390	50.5
ERA OF TRANSPLANTATION		
Era 1 (1983–1992)	175	20.4
Era 2 (1993–2002)	177	20.6
Era 3 (2003–2007)	156	18.2
Era 4 (2008–2013)	188	21.9
Era 5 (2014–2020)	162	18.9
On high-urgency list [y/n]	189	22.1
Pre-existing portal vein thrombosis	21	3.6
Combined pLTx to other organ	21	2.4
Kidney	17	81.0
Lung	3	14.3
Bone marrow	1	4.8
Hepaticojejunostomy as biliary anastomosis	492	63.8
Graft loss due to death or re-transplantation	294	34.8
Blood mismatch	28	3.7
Subsequent re-transplantation	153	17.8
Death after pLTx (of patients, *n* = 705)	141	20.0
Recipient COD: infection	61	43.9
Recipient COD: cerebral event	20	14.4
Recipient COD: cardiopulmonal event	19	13.7
Recipient COD: liver graft related	17	12.2
Recipient COD: malignancy	13	9.4
**Continuous variables**	**Mean** **(Median)**	**Standard deviation** **(Range)**
Age at pLTx	6.03 (4.02)	5.47 (0.05–17.98)
Weight [kg]	22.3 (15.8)	17.99 (2.7–92)
Height [cm]	105.7 (98)	37.7 (45–192)
BMI [kg/m^2^]	16.99 (16.41)	2.95 (11.46–35.34)
Creatinine at pLTx (µmol/L)	52.1 (28)	103.7 (6–815)
Bilirubine at pLTx [µmol/L]	188.5 (103)	209.4 (2–977)
Albumine at pLTx (g/L)	33.4 (33)	8.0 (12–67)
INR at pLTx	1.31 (1.66)	0.87 (0.9–6.4)
Lab-MELD	9.6 (6)	7.66 (6–40)
Ped-MELD (since 2006)	29.3 (28)	5.84 (22–40)
Waiting time for pLTx all status [days]	158.9 (94)	194.4 (1–1141)
Waiting time for pLTx on high urgency list [days]	6.15 (4)	6.22 (1–41)
Waiting time for pLTx (non-HU list) [days]	199.5 (146]	202.5 (1–1141)
ICU stay post pLtx [days]	9 (15)	17.4 (1–153)
NON-ICU stay post Ltx [days]	28 (30)	27.8 (0–295)

**Table 4:** Recipient-specific risk factors affecting patient and graft survival in univariate analysis. For continuous variables, HR is displayed as change per unit of regression.

	PATIENT SURVIVAL			GRAFT SURVIVAL		
Variable	HR	95%-CI	*p*-Value	HR	95%-CI	*p*-Value
**ANTHROPOMETRIC DATA**						
Recipient Age at tx	**1.038**	**1.009–1.068**	**0.0117**	1.002	0.981–1.023	0.8561
Age group < 1 year	**0.645**	**0.399–0.996**	**0.0477**	0.855	0.633–1.137	0.2874
Age group ≥ 1/< 6 years	1.089	0.770–1.525	0.625	1.235	0.972–1.563	0.0838
Age group ≥ 6/< 12 years	0.764	0.485–1.156	0.209	**0.704**	**0.510–0.951**	**0.0214**
Age group ≥ 12 years	**1.627**	**1.120–2.322**	**0.0114**	1.205	0.908–1.577	0.1926
Weight [kg]	1.008	0.999–1.017	0.0581	1.001	0.995–1.008	0.6818
Height [cm]	1.002	0.997–1.006	0.4503	0.999	0.996–1.003	0.7332
BMI [kg/m^2^]	1.030	0.973–1.084	0.2970	0.997	0.956–1.037	0.8941
Sex [female]	0.785	0.586–1.046	0.0990	0.898	0.710–1.134	0.3674
**LABARATORY VALUES**						
Creatinine at pLTx (µmol/L)	1.001	0.996–1.003	0.6528	1.000	0.997–1.002	0.9365
Albumine at pLTx (g/L)	0.991	0.932–1.049	0.7511	0.981	0.950–1.013	0.2446
INR at pLTx	**1.673**	**1.167–2.285**	**0.0007**	**1.370**	**1.085–1.682**	**0.0096**
Bilirubine at pLTx [µmol/L]	**1.002**	**1.001–1.003**	**0.0037**	1.001	1.000–1.002	0.0619
Pediatric MELD-Score	**0.809**	**0.720–0.893**	**<0.0001**	**0.896**	**0.840–0.959**	**0.0004**
**INDICATIONS**						
Biliary atresia	**0.629**	**0.441–0.878**	**0.0059**	0.772	0.588–1.003	0.0525
Kasai procedure in BA	0.655	0.283–1.899	0.3983	0.833	0.411–1.993	0.6523
Acute liver failure	**2.536**	**1.729–3.615**	**<0.0001**	**2.046**	**1.464–2.790**	**<0.0001**
PFIC	**0.457**	**0.194–0.899**	**0.0211**	0.781	0.460–1.235	0.3057
Alagille syndrome	0.698	0.248–1.524	0.401	0.798	0.380–1.458	0.4900
Alpha-1-antitrypsin-deficiency	1.423	0.643–2.701	0.3540	1.542	0.840–2.579	0.1521
Cryptogenic biliary or other cirrhosis	0.662	0.235–1.445	0.3305	0.780	0.372–1.424	0.4452
Cystic fibrosis	0.693	0.246–1.514	0.3909	**0.413**	**0.147–0.896**	**0.0225**
Vascular or other liver disease	0.647	0.199–1.526	0.3559	1.077	0.513–1.968	0.8275
Primary sclerosing cholangitis	1.052	0.324–2.481	0.920	1.286	0.549–2.517	0.5276
Wilson’s disease	1.295	0.460–2.827	0.5837	1.008	0.398–2.066	0.9842
Hepatoblastoma	0.765	0.235–1.816	0.5831	**0.393**	**0.121–0.922**	**0.0293**
Autoimmune hepatitis	0.809	0.134–2.537	0.7580	0.696	0.172–1.820	0.5067
Re-transplantation	**1.781**	**1.274–2.448**	**0.0010**	1.318	0.985–1.737	0.0562
**ERA of TRANSPLANTATION**						
Era 1 (1983–1992)	**3.186**	**2.268–4.451**	**<0.0001**	**2.552**	**1.997–3.245**	**<0.0001**
Era 2 (1993–2002)	**1.466**	**1.009–2.094**	**0.045**	1.262	0.961–1.639	0.0930
Era 3 (2003–2007)	0.808	0.503–1.241	0.3405	0.872	0.635–1.173	0.3731
Era 4 (2008–2013)	**0.347**	**0.186–0.591**	**<0.0001**	**0.584**	**0.415–0.803**	**0.0007**
Era 5 (2014–2020)	**0.073**	**0.012–0.231**	**<0.0001**	**0.246**	**0.137–0.406**	**<0.0001**
Waiting time for Tx [days]	**0.998**	**0.996–0.999**	**0.0002**	**0.999**	**0.998–1.000**	**0.00163**
Waiting time for pLTx [days] non-HU	0.999	0.998–1.000	0.1195	0.999	0.999–1.000	0.0880
On high urgency list [y/n]	**2.160**	**1.600–2.896**	**<0.0001**	**1.686**	**1.231–2.295**	**0.0013**
Waiting time for Tx on HU list [days]	**1.057**	**1.016–1.091**	**0.0089**	1.018	0.984–1.047	0.2832
Pre-transplant portal vein thrombosis	1.992	0.899–3.786	0.0570	1.575	0.781–2.805	0.1593
Blood mismatch	1.409	0.964–2.002	0.0753	1.204	0.869–1.632	0.2563
Sex mismatch	0.805	0.604–1.072	0.1367	0.847	0.671–1.069	0.1618
Biliodigestive anastomosis	**0.735**	**0.546–0.994**	**0.0462**	0.826	0.647–1.059	0.1311
Subsequent re-Tx	**1.934**	**1.400–2.637**	**<0.0001**	n/a	n/a	n/a
ICU stay	**1.010**	**1.004–1.017**	**0.0041**	**1.009**	**1.003–1.014**	**0.0029**
Non-ICU stay	**0.960**	**0.952–0.968**	**<0.0001**	**0.970**	**0.963–0.976**	**<0.0001**
Combined liver transplant with other organ	0.801	0.198–2.105	0.6923	0.690	0.213–1.618	0.4328

**Table 5:** Independent significant recipient-specific risk factors for patient survival in multivariate analysis. For continuous variables, HR is displayed as change per unit of regression. As there was a sub-analysis of the different eras of transplantation, no overall hazard ratio could be calculated. Model: LogLikelihood: 592.24; Chi2:66.086; *p* < 0.0001.

Variable	Log-Ranking	Hazard Ratio	95%-CI	*p*-Value
Era of pLTx [1,2,3,4,5]	7378	n/a	n/a	<0.00001
Waiting time on high urgency list [d]	2588	1.073	1.028–1.112	0.00258
Indication: Acute liver Failure	1944	2.015	1.181–3.296	0.01139
Subsequent re-transplantation	1755	1.744	1.106–2.674	0.01757
Indication: Biliary atresia	1502	0.575	0.332–0.954	0.03151

**Table 6:** Independent significant risk factors for graft survival in multivariate analysis. For continuous variables, HR is displayed as change per unit of regression. As there was a sub-analysis of the different eras of transplantation, no overall hazard ratio could be calculated.

Variable	Log-Ranking	Hazard Ratio	95%-CI	*p*-Value
Era of pLTx	9657	n/a	n/a	<0.0001
Indication: Acute liver failure	3923	1.935	1.364–2.677	00001
Pretransplant portal vein thrombosis	1482	2.016	1.009–3.643	0.0330

## Data Availability

Data in the study can be obtained from the corresponding author upon reasonable request.
